# The Guidance of Attentional Selectivity in Visual Search Is Always Feature‐Based: Behavioral and Electrophysiological Evidence From Feature and Conjunction Search Tasks

**DOI:** 10.1111/psyp.70169

**Published:** 2025-10-22

**Authors:** Ziyi Wang, Mikel Jimenez, Martin Eimer, Anna Grubert

**Affiliations:** ^1^ Department of Psychology Durham University Durham UK; ^2^ Departamento de Psicología Básica I Universidad Nacional de Educación a Distancia Madrid Spain; ^3^ Department of Psychological Sciences, Birkbeck University of London London UK

## Abstract

It is generally assumed that the guidance of attention in visual search operates in a feature‐based fashion, but this conclusion is mainly based on the results of search tasks with feature‐defined targets. Here, we investigated whether attentional templates may contain integrated object‐based representations in tasks where targets are defined by feature conjunctions. Attentional load was manipulated by instructing participants to search for a single or one of two different possible targets. Search targets were either defined by a particular color or shape (feature search tasks) or by a color/shape combination (conjunction search task), and load effects were compared between these two types of tasks. Load effects on search performance as well as on electrophysiological markers of attentional guidance (N2pc components) and the number of attentional templates activated in working memory (CDA components) were much more pronounced during conjunction search. This suggests that attentional templates are always feature‐based, even when object‐based templates are in principle available to reduce memory load.

## IMPACT STATEMENT

This study provides decisive evidence that attentional templates guiding visual search are fundamentally feature‐based, even in conjunction search tasks in which object‐based representations might be beneficial. By combining behavioral and electrophysiological measures of search efficiency and memory load, the study challenges the assumption that working memory capacity for integrated objects extends to attentional guidance. These findings refine existing models of visual search and advance understanding of the interactions between visual attention and working memory.

## 1 Introduction

Attentional guidance toward task‐relevant objects is controlled by attentional templates, that is, target representations held in working memory (e.g., Duncan and Humphreys 1992; Olivers et al. 2011; Soto et al. 2008). Attentional templates contain target descriptions, such as a particular color or shape, which are activated in a preparatory (e.g., Chelazzi et al. 1998) and transient fashion (e.g., Grubert and Eimer 2018) before the arrival of the search display. Once activated, any object in the visual field that matches the content of the current attentional template will automatically attract attention (e.g., Desimone and Duncan 1995; Folk et al. 1992; Grubert and Eimer 2016a; Martinez‐Trujillo and Treue 2004; Wolfe 2021) in a spatially global manner (e.g., Berggren et al. 2017; Eimer 2014; Jimenez et al. 2024; McAdams and Maunsell 2000; Saenz et al. 2002). However, the content of attentional templates remains a topic of debate. One controversial issue is concerned with the question as to whether attentional templates contain single features per default (e.g., blue), or whether they can contain feature conjunctions (e.g., blue and large) that form an integrated object representation. Such templates would clearly be useful for the guidance of search for conjunctively defined targets, e.g., when searching for a large blue textbook between other blue but small or large but green books on the bookshelf.

Contemporary theories of visual search propose that the initial analysis of the visual field is feature‐based (Bundesen 1990; Huang and Pashler 2007; Itti and Koch 2001; Müller and Krummenacher 2006; Wolfe 2021). According to Guided Search 6.0 (Wolfe 2021), attentional deployment is controlled by a feature‐based “priority map” which signals the most likely target location, and which is continuously updated during visual search as stimulus‐, user‐, and context‐dependent parameters change. Only when a potential target location is selected, object features are bound together, and holistic object representations are matched with stored target representations held in long‐term memory for target identification (see also Cunningham and Wolfe 2014). Behavioral (e.g., Becker et al. 2020; Dent 2023), eye tracking (e.g., Williams and Reingold 2001), computational modeling (e.g., Rutishauser and Koch 2007), and electrophysiological studies (e.g., Berggren and Eimer 2018; Eimer and Grubert 2014) have delivered empirical evidence for the hypothesis that early visual selection is feature‐based even during conjunction search, when multiple task relevant features are presented in the same object.

Clear evidence for feature‐based attentional guidance during conjunction search comes from a study from our own lab (Eimer and Grubert 2014), where participants searched for conjunctive color and shape targets (e.g., blue square). In some trials, the target was absent, but a color‐matching (e.g., blue circle) or a shape‐matching (e.g., yellow square) distractor was presented. In that study, we recorded EEG and measured the N2pc component of the event‐related potential as an electrophysiological marker of attentional selection in response to such targets and partially matching distractors. The N2pc is an increased negativity, elicited at around 200 ms after stimulus onset at posterior electrode sites over extrastriate visual cortex (e.g., Hopf et al. 2000), contralateral to the side of an attended object (Eimer 1996; Luck and Hillyard 1994; Woodman and Luck 1999). It is believed to reflect the initial allocation of attention to possible target objects in visual search and may also be sensitive to processing stages that follow object selection (see the General Discussion for further details). All objects that contained any of the two target features, that is, targets, color‐matching and shape‐matching distractors, produced reliable N2pcs, indicating that attentional selection was guided by individual target features as opposed to integrated object representations (in which case partially matching distractors should have been ignored since they never matched the full target representation). Interestingly, the target N2pc was initially identical to the summed N2pc of the partially matching distractors, but after approximately 250 ms, the target N2pc became larger than the summed distractor N2pc. This finding suggests that attention was initially guided by signals from the color and shape dimension independently, before becoming sensitive to the joint presence of both relevant target‐defining features within the same object.

Since attentional templates are believed to be held in working memory (e.g., Duncan and Humphreys 1992; Olivers et al. 2011; Soto et al. 2008), the question arises why such templates should not also represent feature conjunctions, as this would be an effective method of reducing working memory load during information retention (e.g., Cowan 2001; Delvenne and Bruyer 2004; Olson and Jiang 2002; Quinlan and Cohen 2011). Evidence for the existence of integrated representations of feature conjunctions in working memory comes from experiments by Luck and Vogel (1997; see also Vogel et al. 2001). They used a change detection task where participants memorized arrays of two, four, or six colored bars of different orientations to detect a change in a subsequent test array. In the single feature conditions, participants were instructed to only memorize the color or orientation of the bars, while they had to memorize both features in the conjunction condition. If only individual features can be held in working memory, memory load would be higher in the conjunction condition, resulting in performance decrements relative to the single feature conditions. However, results revealed identical accuracy rates in both the single feature and the conjunction conditions across all set sizes. This shows that participants successfully integrated the color and orientation features into a coherent object representation and kept working memory load identical in the conjunction and single feature tasks. Given these observations, it is puzzling that attentional templates in working memory should not also represent combinations of features during conjunction search. Previous search studies may have failed to find evidence for conjunctively defined attentional templates because they investigated search for a single target object and thus most likely did not exceed working memory capacity limits. It might be possible that with an increased working memory load, observers may have a stronger incentive to integrate single features into object‐based templates for visual search (see Berggren and Eimer 2018, for initial electrophysiological evidence for this during search for two feature conjunctions, and Balaban and Luria 2016, for evidence that feature integration in working memory, as measured in change detection tasks, also depends on stimulus parameters and task demands).

In the present study, we tested this possibility by contrasting single feature search tasks where targets were defined by colors or shapes, and a conjunction search task in which targets were defined by combinations of color and shape. Critically, we also manipulated working memory load in both tasks by instructing participants to either search for a single constant target (low load) or one of two equally possible targets (high load).

The relevant target features for each trial were cued prior to search display onset. Load effects (costs associated with the activation of two as compared to one attentional template) were substantiated at consecutive stages of the search process, i.e., during template activation prior to search, during attentional guidance, and at response selection and execution.

Several studies, using a variety of search paradigms and measures, have shown that two color‐specific attentional templates can be activated in parallel when observers search for either of two possible color defined targets (e.g., Barrett and Zobay 2014; Beck et al. 2012; Berggren and Eimer 2019; Christie et al. 2015; Grubert et al. 2016; Grubert and Eimer 2016a, 2023; Irons et al. 2012; Kristjánsson and Kristjánsson 2018; Moore and Weissman 2010; Ort et al. 2019). Importantly, these studies revealed substantial behavioral costs in such two‐color search tasks as compared to single‐color search, as well as small but systematic delays of N2pc components triggered in response to search target objects. The behavioral costs are assumed to be mainly generated at stages of attentional selectivity that follow the initial guidance of attention by attentional templates (e.g., Ort et al. 2019; and Ort and Olivers 2020). In contrast, the target N2pc delays observed in two‐color search tasks show that attentional guidance is also affected, possibly due to mutual inhibition between simultaneously activated attentional templates (e.g., Grubert et al. 2025; Kerzel and Grubert 2022; Ort et al. 2019). In the present study, we compared these costs associated with having to search for either of two possible target objects relative to single object search between tasks where targets were defined by a single feature (either color or shape) and a task where they were defined by a color/shape conjunction. If attentional templates can hold integrated target representations, load effects as measured at the behavioral and electrophysiological level should be identical in single feature and conjunction search. Alternatively, if attentional templates can only hold single features, load effects should be substantially increased in the conjunction task, because the feature load is effectively doubled relative to the feature search tasks.

In addition to using the N2pc as a measure of load‐induced costs on the guidance of attention by target templates, we also employed an additional electrophysiological marker (contralateral delay activity, CDA) to directly assess the number of attentional templates that are held in low‐load and high‐load trials in the feature and conjunction search tasks. The CDA component is a sustained negative deflection triggered at posterior electrode sites which is elicited during the active maintenance of target representations in working memory (e.g., Carlisle et al. 2011), which increases in size with increasing memory load (e.g., Vogel and Machizawa 2004). In a previous experiment from our lab (Grubert et al. 2016), we measured CDA components during the period prior to the presentation of search displays when observers had to search for one, two, or three possible color‐defined targets. CDA components systematically increased in amplitude from one‐ to two‐ and then again from two‐ to three‐color search, indicating that participants did activate one, two, or three color templates prior to search. In the present study, we measured CDAs during the period before search display onset, separately for low‐load and high‐load trials in the feature and conjunction search tasks. Larger CDAs were again expected for high‐load trials, but the critical question was whether these load effects would be more pronounced in the conjunction search task (as predicted by the hypothesis that attentional templates are always feature‐based), or similar in size across all tasks (indicative of integrated object‐based templates during conjunction search).

All these hypotheses were explicitly tested in Experiment 2. The goal of Experiment 1 was to establish whether behavioral and electrophysiological costs associated with increased load during search for two possible targets as compared to a single target are equivalent in tasks where targets are defined by color or by their shape. This is important because most of the previous studies that investigated such feature‐based load effects employed color‐defined targets. Furthermore, there is behavioral (e.g., Moutoussis and Zeki 1997; Wolfe and Horowitz 2004) and electrophysiological (e.g., Lee et al. 2018) evidence that the attentional guidance by color may have priority over shape‐based guidance. And these experiments have support from historical anatomical studies (e.g., Barlow 1972) as well as more contemporary fMRI studies which found clear segregation between color and shape processing in the brain (e.g., Lafer‐Sousa et al. 2016). These findings suggest that there might be qualitative differences between color and shape templates, and that it might be possible that the parallel guidance by multiple shape templates cannot be implemented as effectively as the guidance by multiple color templates. If there were systematic differences in the size or timing of these effects when targets are defined by their shape rather than their color, this would complicate any interpretation of load effects during color/shape conjunction search. Therefore, Experiment 1 only included tasks where observers searched for one or two possible targets, and these targets were feature‐defined (either by color or shape).

## 2 Experiment 1

### 2.1 Methods

#### 2.1.1 Participants

Eighteen participants were tested in Experiment 1 and received £10 per hour for their work. The experiment was approved by the Ethics Committee of the Psychology Department at Durham University and was conducted in accordance with the Declaration of Helsinki. Participants gave informed written consent prior to testing. Three participants were excluded from data analysis, two of them because of excessive eye movements resulting in a loss of more than 40% of all trials during artifact rejection (a priori criterion to maintain a minimum of 160 trials per N2pc), and the other one because of accuracy rates below 80%. Of the remaining 15 participants, aged between 19 and 45 years (*M* = 26.5, SD = 5.9), ten were female and five were male. Three of the 15 participants were left‐handed and 12 were right‐handed. All participants had normal or corrected‐to‐normal vision and full color vision, as verified with the Ishihara color vision test (Ishihara 1918). The sample size of 15 was calculated by means of an a priori power analysis using MorePower 6.0.1 (Campbell and Thompson 2012) to detect a 2 × 2 interaction (memory load × laterality) in a repeated‐measures ANOVA (within‐subjects) with an assumed alpha of 0.05, power of 0.95, and a large effect size > 1.^1^

#### 2.1.2 Stimuli and Procedures

Participants were seated in a sound attenuated and dimly illuminated Faraday cage. Visual stimuli were presented on a PC monitor at a viewing distance of approximately 90 cm. A 17‐in. Samsung wide Syncmaster 753S monitor (1280 × 1024 pixels resolution, 100 Hz refresh rate) was employed. Stimulus presentation, timing, and response collection were controlled by an LG Pentium PC running under Windows XP, using the Cogent 2000 toolbox for MATLAB.

**FIGURE 1:**
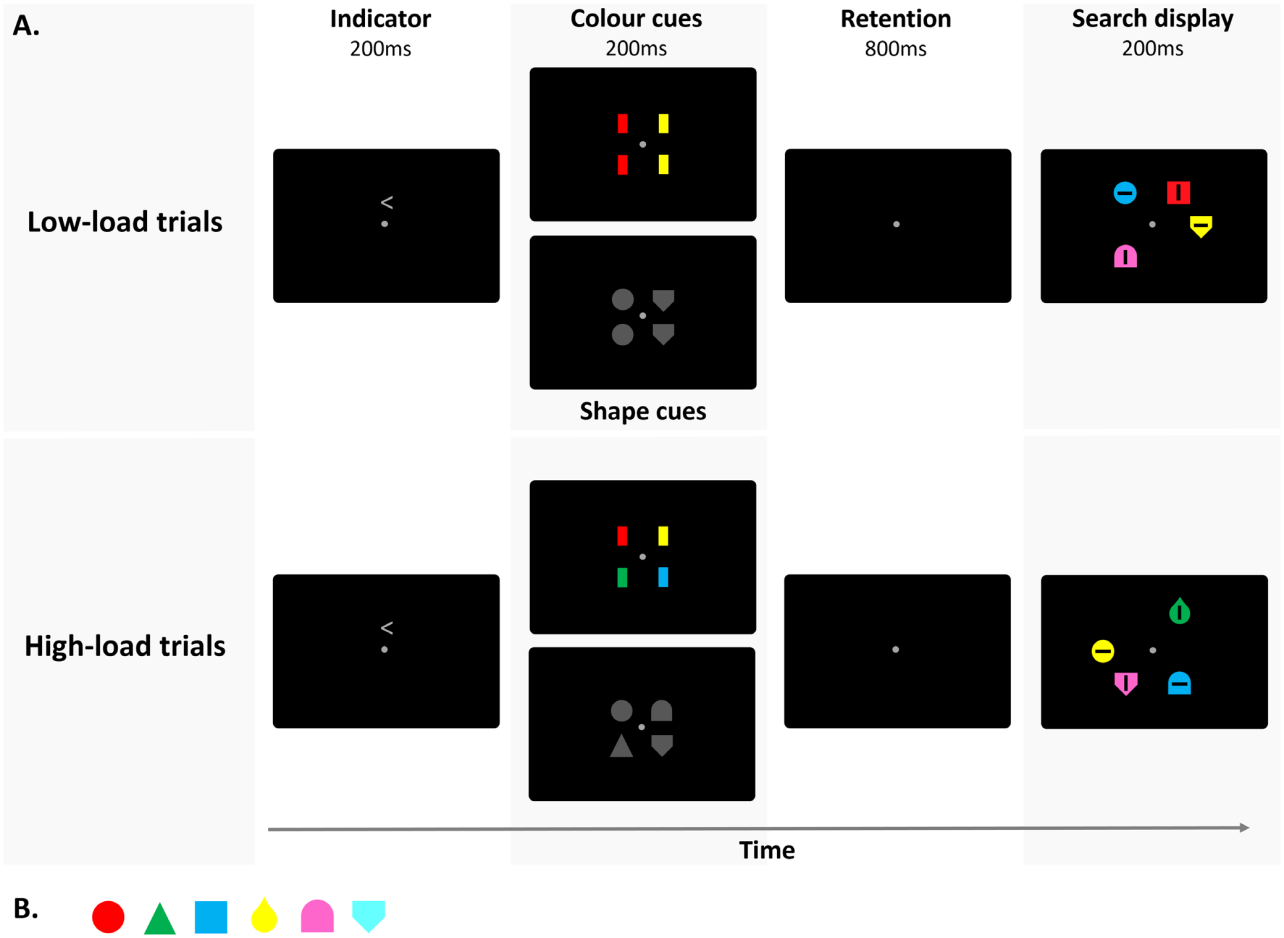
Schematic illustration of the stimuli and temporal trial sequence in Experiment 1. Each trial began with the presentation of an arrowhead pointing to the left or right to indicate the task‐relevant side for the upcoming cue display. Cues informed participants about the upcoming one (low‐load trials) or two (high‐load trials) target colors (color task) or shapes (shape tasks), respectively. Cue displays were followed by a retention period and the search displays, which contained four differently colored shapes randomly presented at four of six possible equidistant positions on an imaginary circle (two in each hemifield). Participants' task was to report the line orientation (horizontal, vertical) shown within the target object that matched the cued target color or shape, respectively.

Stimuli were presented on a black background. A gray fixation point was presented at the center of the screen throughout each experiment block (0.2° × 0.2° of visual angle; x/y CIE color coordinates: 0.317/0.359). Each trial started with the presentation of an indicator display for 200 ms, which was followed by a 200 ms cue display. After an 800 ms blank retention period, the search display appeared for 200 ms (Figure 1A). The inter‐trial interval between the offset of a search array and the onset of the indicator display of the next trial was 2000 ms.

Two blocked task conditions were tested in Experiment 1. In the *color task*, participants searched for a predefined target‐color object among differently colored nontargets. In the *shape task*, they searched for a predefined target shape among different nontarget shapes. Importantly, search displays were physically identical in the two task conditions as they each contained four differently colored individual shapes (0.5° × 0.5°) with a black horizontal (0.4° × 0.1°) or vertical (0.1° × 0.4°) line inside. Each search array contained two objects at each display side. These were presented randomly at two out of three possible locations in the right (1, 3, or 5 o'clock positions of an imaginary clock face) or left hemifield (7, 9, or 11 o'clock positions). All stimulus locations had an eccentricity of 1° from central fixation. There were six possible stimulus colors (Figure 1B): red (x/y CIE color coordinates: 0.621/0.335), green (0.285/0.616), blue (0.180/0.176), yellow (0.462/0.465), pink (0.340/0.174), and cyan (0.217/0.355). All colors, including the gray of the fixation point, were equiluminant (~9.5 cd/m^2^). There were also six possible stimulus shapes (Figure 1B): circle, triangle, square, drop, gate, and inverted house. The drop, gate, and inverted house shapes were created by combining the canonical shapes, mimicking the mixing of the three primary colors (to achieve yellow, pink, and cyan). All shapes were matched with respect to size in pixels (36 × 36).

One of the four objects in each search display was characterized by the cued target color or shape, respectively, and participants' task was to find this search target and report the orientation (horizontal, vertical) of the black line inside it. Each color/shape combination was used equiprobably as the target within each block, even though the object shape was task irrelevant in the color task, and vice versa, the object color was irrelevant in the shape task. The target location was pseudo‐randomized so that, in each block, the target appeared equiprobably in the left and right hemifield (the exact location within each hemifield was fully randomized). The line orientations within the four search objects were independently and fully randomly selected in each trial.

The target identity was cued prior to search onset and changed in every trial. Cue displays contained two sets of two vertically aligned objects presented bilaterally to the left and right of fixation at a horizontal distance of 0.3° (measured relative to the center of each cue item). In each hemifield, the two cues were presented at a vertical distance of 0.3° above and below the horizontal midline. In the color task, the cues were rectangles (0.2°x0.4°) shown in the colors described above. In the shape task, the cue displays contained the shapes described above in gray color (0.4° × 0.4°). Only one side of the cue display was task relevant in each given trial and contained the target colors/shapes for the upcoming search. The relevant cue side was randomized but equiprobable in each block and was determined at the beginning of each trial by means of an indicator display. Indicator displays contained a central gray arrowhead presented above fixation, which pointed toward the side with the task relevant target color/shape cues. In 50% of the trials, the target color/shape cues and the subsequent target appeared on the same side, in the other 50%, they were presented in opposite hemifields. Each task condition (color, shape task) contained two equiprobable and intermixed memory load conditions. In *low‐load trials*, the two target cues were identical and showed the same single target color or shape, respectively. In *high‐load trials*, the two target cues showed two different colors or shapes, respectively, either of which could equally likely appear in the search display. Note that there was only ever one target in each search display. The other cued target color/shape never appeared in the search display. The location of the cue (top/bottom) that contained the actual target color in each trial was fully randomized between trials. The nontarget cues on the task irrelevant side of the cue displays randomly contained one (low‐load trials) or two (high‐load trials) different colors or shapes, respectively, which always appeared as nontargets in the search display, too.

Responses were given bi‐manually on a standard keyboard using the up and down arrow keys, respectively. Response‐to‐key (top or bottom key for horizontal or vertical line) and hand‐to‐key assignments (left or right hand on top or bottom arrow key) were counterbalanced across participants but remained the same for each participant throughout the experiment. Each block contained six trials for each combination of memory load (low‐, high‐load), cue side (left, right hemifield) and target side (left, right hemifield). Both the color and shape task each contained 12 blocks of 48 trials, resulting in a total of 1152 experimental trials for Experiment 1. Seven of the participants completed the color task first and then the shape task, and vice versa for the other eight participants. Before the first block of each task, participants completed a practice block to familiarize themselves with the task. These practice data were not recorded.

#### 2.1.3 EEG Recording and Data Analyzes

The continuous EEG was DC‐recorded from 23 scalp electrodes (EasyCap, Brain Products) at standard positions of the extended 10/20 system (Fpz, F7/8, F3/4, Fz, FC5/6, T7/8, C3/4, Cz, CP5/6, P7/8, P3/4, Pz, PO7/8, Oz), and two HEOG electrodes placed at the outer canthi of the eyes. EEG data were recorded using the Brain Vision Recorder (BrainAmp DC amplifier, Brain Products GmbH, Gilching, Germany) at a sampling rate of 500 Hz with a 40 Hz low‐pass filter (causal Butterworth filter, 12 dB/octave). No additional offline filters were applied after data acquisition. Impedances were kept below 5 kΩ. All channels were referenced online to the left‐earlobe and re‐referenced offline to an average of both earlobes. All EEG data processing was conducted with the BrainVision Analyzer software (Brain Products GmbH, Gilching, Germany). Data from trials with incorrect or missing responses, or with anticipatory (< 200 ms) or slow responses (> 1500 ms), were not included in the analysis. Trials contaminated with eye movements (exceeding ±30 μV in the bipolar HEOG channel), eye blinks (exceeding ±60 μV at Fpz), and muscular movement artifacts (exceeding ±80 μV in all other channels) were removed as artifacts. Artifact rejection resulted in an exclusion of 13.8% of all trials in the color task (SD = 15.2%; ranging between 0.6% and 33.9% across participants) and 12.5% of all trials in the shape task (SD = 11.8%; ranging between 0.9% and 35.3% across participants). The remaining EEG was segmented into 500 ms epochs ranging from 100 ms before to 400 ms after search display onset. The first 100 ms served as a pre‐stimulus baseline. EEG was averaged separately for each combination of memory load (low‐, high‐load) and target side (left, right hemifield). N2pc mean amplitudes were quantified at lateral posterior electrodes PO7 and PO8, contralateral and ipsilateral to the target side, in the 190–290 ms interval after search display onset. N2pc onset latencies were calculated based on difference waveforms, computed by subtracting ipsilateral from contralateral activity. Jackknife‐based procedures were applied (Miller et al. 1998) in which 15 grand‐average difference waves were computed, always leaving out one different participant from the sample. N2pc onset latencies were defined as the point in time when the ascending flank of each subsample difference wave reached −0.8 μV (50% of the peak amplitude of the averaged target N2pc, pooled across all targets in all conditions of Experiment 1; see e.g., Grubert and Eimer 2023, for similar procedures). To ascertain that any N2pc latency differences between conditions obtained in these analyses were not limited to the use of a fixed onset criterion, we also calculated fractional N2pc peak latencies. These were defined as the time point at which the ascending flank of the jack‐knifed waveform reached 50% of its peak amplitude. All jack‐knifed *F*‐values (labeled *F*_*c*_) were power‐corrected according to Ulrich and Miller (2001). Effect sizes are reported as Cohen's *d* (Cohen 1988) with a CI of 95% for *t*‐tests, and as partial eta squared ($\eta_{p}^{2}$) for *F*‐tests ($\eta_{\mathrm{pc}}^{2}$ for power‐corrected *F*‐testes, respectively; see Grubert and Eimer 2016b, for detailed procedures). When necessary, *F*‐tests were Greenhouse–Geisser corrected and *t*‐tests were Bonferroni corrected. All *t*‐tests were two‐tailed. Bayesian statistics (Rouder et al. 2012) were used to provide additional statistical evidence. Bayesian ANOVAs were based on Bayesian Model Averaging (Wagenmakers et al. 2018; Hoeting et al. 1999) and were computed across matched models (i.e., to compare a model with a specific effect to the equivalent model without that effect; reported as *BF*_*incl*_). Bayesian *t*‐tests are report as *BF*_*10*_. Substantial evidence for the alternative hypothesis is marked by Bayes factors *BF* > 3 (Jeffreys 1961), indicating that the empirical data are more than three times more likely under the alternative hypothesis than the null hypothesis. For Bayesian statistics on jack‐knifed data, estimates of each participant's latency were retrieved from the subaverage scores using the formula as suggested by Smulders (2010), to avoid an inflated signal‐to‐noise ratio. All statistical analyses, including Bayesian statistics were conducted with JASP statistical software (version 0.18.1.0).

### 2.2 Results

#### 2.2.1 Behavioral Results

**FIGURE 2:**
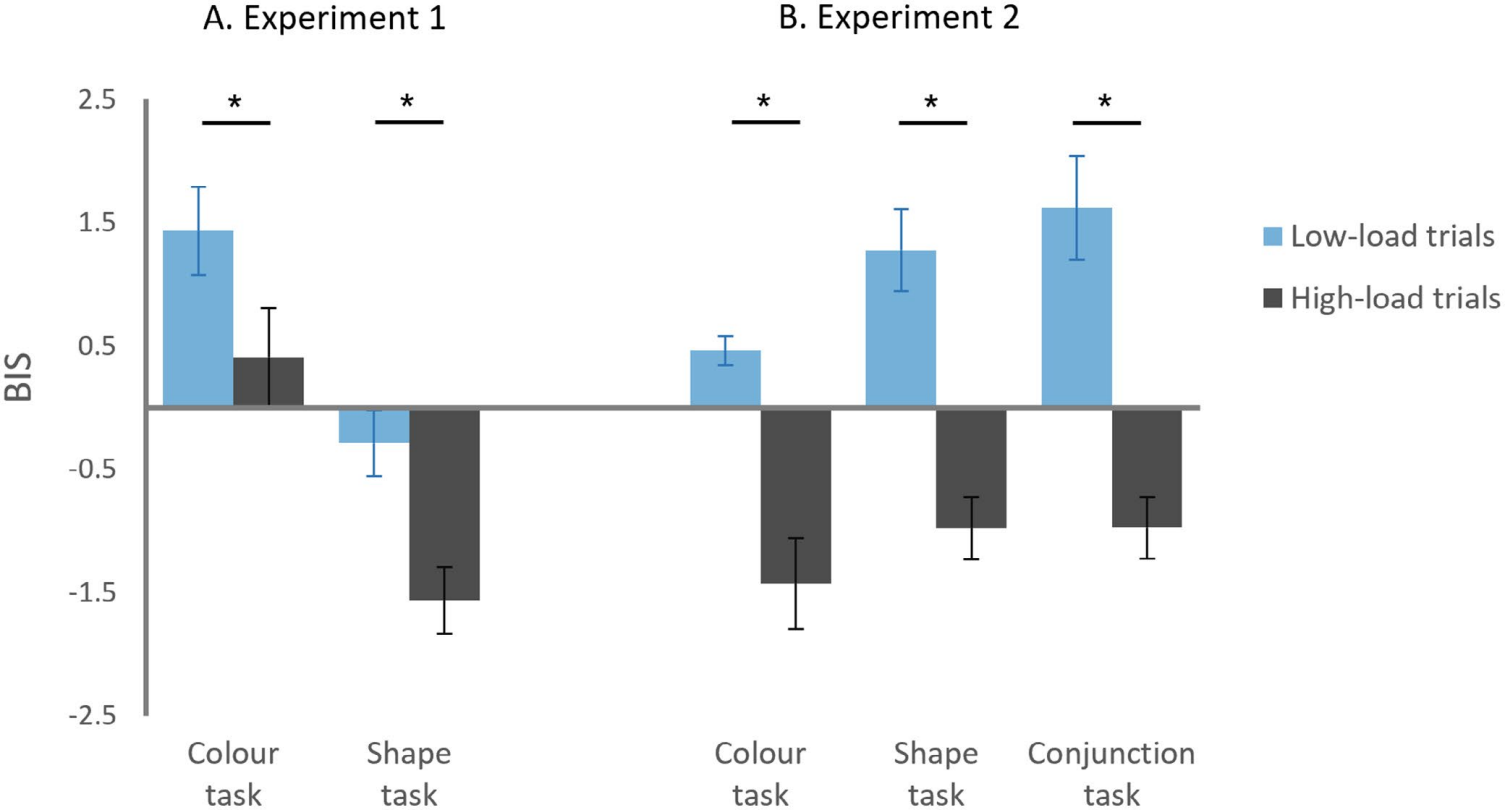
Balanced Integration (BI) scores measured in low‐ and high‐load trials in the color and shape tasks of Experiment 1 (A) and the color, shape, and conjunction tasks of Experiment 2 (B). BI scores reflect relative performance differences, taking both RTs and error rates into account at the same time. Higher BI scores indicate more efficient task performance than lower BI scores. Error bars indicate standard errors of the mean. Statistically reliable load effects (differences between high‐ and low‐load trials) are marked by asterisks.

Trials with anticipatory (< 200 ms) and slow (> 1500 ms) responses were excluded from analysis (2.7% of all trials). Behavioral data are presented as Balanced Integration scores (BI scores; Figure 2, left panel) to account for a speed‐accuracy trade‐off (see mean RTs and error rates in the Table S1). BI scores account for speed‐accuracy trade‐offs by subtracting the standardized mean RT from the standardized mean accuracy rates (Liesefeld et al. 2015; Liesefeld and Janczyk 2019). BI scores must be understood to reflect relative performance differences, that is, whether one condition is more or less difficult than the other. BI scores > 0 indicate performance above average, while BI scores < 0 reflect below average performance. Therefore, a relatively higher BI score means that participants performed “better” in that condition than another, taking both, RTs and error rates into account at the same time. Mean BI scores were subjected to a repeated‐measures ANOVA with the factors task (color, shape) and memory load (low‐, high‐load). A main effect of task revealed larger BI scores in the color (0.92) than shape task (−0.93), *F*(1,14) = 14.6, *p* = 0.002, *η* ^2^_*p*_ = 0.51, indicating that selection was more efficient in the color task. BI scores were also larger in low‐load (0.57) than high‐load trials (−0.58), *F*(1,14) = 49.2, *p* < 0.001, *η* ^2^_*p*_ = 0.78, reflecting higher performance levels when participants searched for one as opposed to two possible target colors or shapes, respectively. There was no significant interaction between these two factors, *F*(1,14) = 1.2, *p* = 0.284, *η* ^2^_*p*_ = 0.08, which shows that load effects (difference between low‐ and high‐load BI scores) were identical in the color (1.84) and shape tasks (1.85). Bayesian Model Averaging also provided clear evidence for the main effects of task (*BF*_*incl*_ = 14.1), and load (*BF*_*incl*_ > 100), and the absence of a task × load interaction (*BF*_*incl*_ = 0.5).

#### 2.2.2 N2pc Results

**FIGURE 3:**
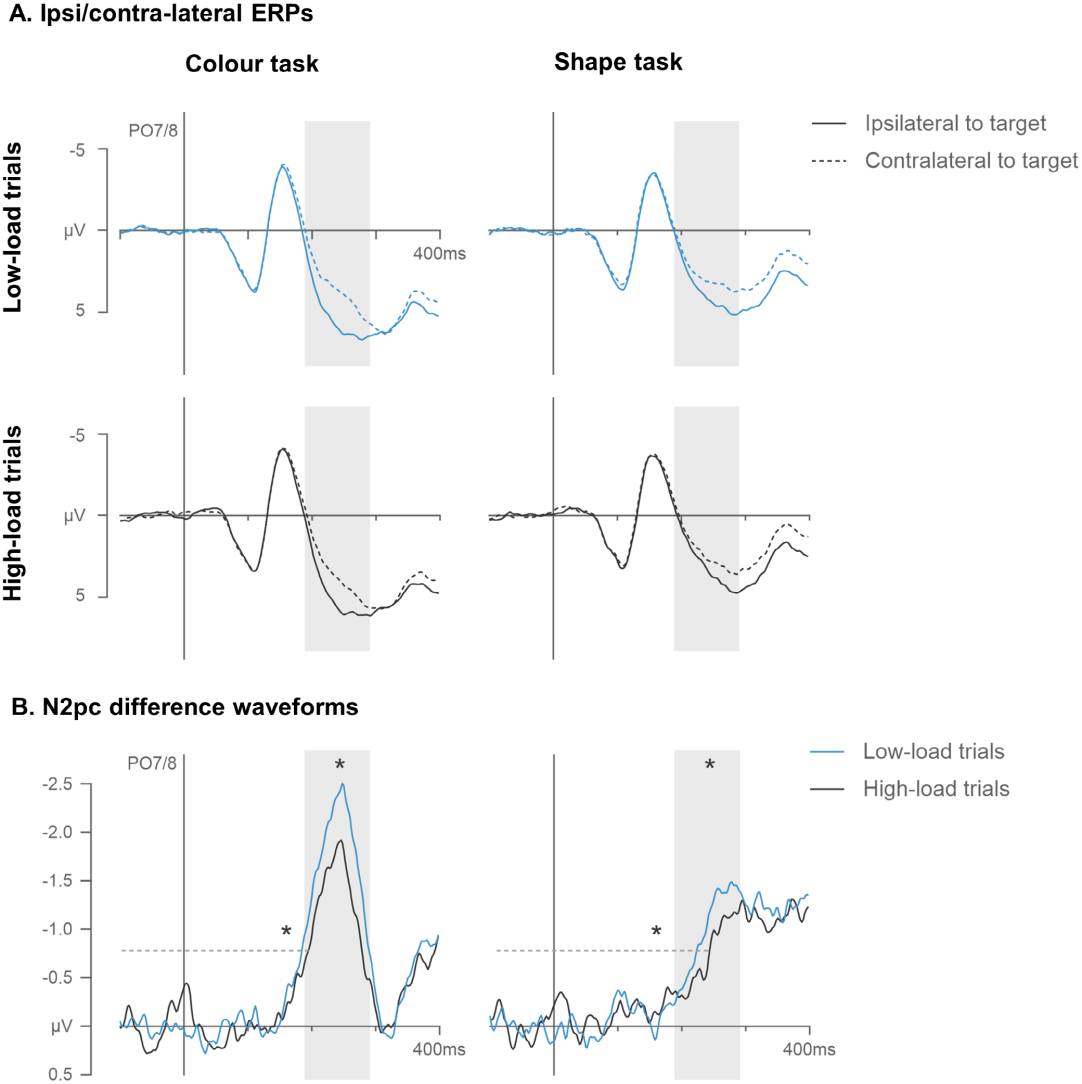
Grand‐averaged ERPs measured at electrodes PO7/8 contralateral and ipsilateral to the targets in low‐ and high‐load trials of the color and shape tasks in Experiment 1 (A). The respective difference waves (contra‐ minus ipsilateral ERPs) are shown below (B). The shaded areas mark the N2pc time window (190–290 ms after search display onset) and the dashed lines indicate the onset latency criterion (−0.8 μV). Asterisks mark statistically reliable load effects (differences between high‐ and low‐load trials) measured in N2pc mean amplitudes (asterisks above amplitudes) and onset latencies (asterisks above onset criterion).

Figure 3A displays grand‐averaged event‐related potentials (ERPs) recorded at electrode sites PO7/8 contralateral and ipsilateral to targets in low‐ and high‐load trials of the color and shape tasks. Clear N2pc components were triggered in the 190–290 ms time interval after search display onset in all four conditions. From the corresponding N2pc difference waves (Figure 3B), it can be seen that N2pcs in the shape, as compared to the color task, were delayed and attenuated. However, memory load effects, i.e., attenuated and delayed N2pcs in high‐ as compared to low‐load trials, appeared to be comparable between the two task conditions.

These observations were confirmed by repeated‐measure ANOVAs on N2pc mean amplitudes with the factors task (color, shape), memory load (low‐, high‐load), and laterality (electrode contralateral, ipsilateral to the target). The ANOVA revealed a main effect of laterality, *F*(1,14) = 59.7, *p* < 0.001, *η* ^2^_*p*_ = 0.81, which interacted with task, *F*(1,14) = 28.0, *p* < 0.001, *η* ^2^_*p*_ = 0.67, and load, *F*(1,14) = 11.1, *p* = 0.005, *η* ^2^_*p*_ = 0.44, confirming that N2pc components were larger in the color (−1.6 μV) than the shape task (−0.9 μV), and in low‐load (−1.4 μV) as compared to high‐load trials (−1.0 μV). Most importantly, there was no three‐way interaction *F*(1,14) = 1.5, *p* = 0.238, *η* ^2^_*p*_ = 0.10. A complementary Bayesian ANOVA also provided evidence for a main effect of laterality (*BF*_*incl*_ > 100), a Laterality × Task interaction (*BF*_*incl*_ = 97.3), and a Laterality × Load interaction (*BF*_*incl*_ = 4.0), but no substantial evidence for the three‐way interaction (*BF*_*incl*_ = 0.4). This suggests that the amplitude load effects (low‐ minus high‐load amplitudes) were comparable between the color (−0.5 μV) and shape tasks (−0.3 μV).

N2pc onset latencies measured with a fixed onset criterion at −0.8 μV were subjected to a repeated‐measure ANOVA with the factors task (color, shape) and memory load (low‐, high‐load), which produced main effects of task, *F*_*c*_(1,14) = 66.3, *p* < 0.001, *η* ^2^_*pc*_ = 0.83, and load, *F*_*c*_(1,14) = 7.0, *p* = 0.007, *η* ^2^_*pc*_ = 0.33, but no reliable interaction, *F*_*c*_(1,14) < 1, *p* = 0.499, *η* ^2^_*pc*_ = 0.03. In other words, N2pc onset latencies were delayed in the shape (234 ms) than color search (192 ms), and in high‐load (221 ms) as compared to low‐load trials (206 ms). But in line with the behavioral and mean amplitude results, the load effects (high‐ minus low‐load latencies) were comparable between the color (11 ms) and shape tasks (19 ms). These interpretations were generally supported by a Bayesian ANOVA which also produced a main effect of task (*BF*_*incl*_ > 100), a marginally significant main effect of load (*BF*_*incl*_ = 2.1), and no reliable interaction (*BF*_*incl*_ = 0.4). To ensure that these onset latency differences were not limited to the use of a fixed onset criterion, we ran a complementary analysis on fractional (50%) peak latencies. Essentially the same results were obtained. N2pc latencies were delayed in the shape (233 ms) as compared to the color task (200 ms), *F*_*c*_(1,14) = 36.0, *p* < 0.001, *η* ^2^_*pc*_ = 0.72, *BF*_*incl*_ > 100, and in high‐load (221 ms) as compared to low‐load trials (212 ms), *F*_*c*_(1,14) = 6.2, *p* = 0.026, *η* ^2^_*pc*_ = 0.31, *BF*_*incl*_ = 2.5 (marginal at the Bayesian level). However, load effects were comparable between the color (5 ms) and shape tasks (13 ms), *F*_*c*_(1,14) = 1.3, *p* = 0.271, *η* ^2^_*pc*_ = 0.09, *BF*_*incl*_ = 0.7.

### 2.3 Discussion of Experiment 1

Experiment 1 revealed clear memory load costs, with lower BI scores and attenuated and delayed N2pc components in high‐load as compared to low‐load conditions.

The load costs observed in the color task mirrored findings of previous studies (e.g., Grubert and Eimer 2013, 2023; Grubert et al. 2025, 2016; Ort et al. 2019). N2pc amplitudes and latencies were attenuated and delayed, respectively, in high‐load as compared to low‐load trials when participants had to activate two as compared to one color template for search.

However, the N2pc delays were small (11 ms), indicating that two color templates were activated simultaneously, as a serial switch between two sequentially activated templates would have taken much longer (e.g., 250–300 ms; Dombrowe et al. 2011). Importantly, Experiment 1 also found that the load costs observed with shape‐defined targets were qualitatively similar to those found for color‐defined targets. There were numerical differences, but statistically, the costs associated with the increased memory load on N2pc amplitudes and latencies were comparable in the color and shape tasks (−0.5 μV vs. −0.3 μV; 11 ms vs. 19 ms). This suggests that the processing costs associated with multiple template activation and between‐template inhibition on feature‐based attentional guidance are equivalent across color and shape. There was, however, a general main effect of task on behavior and target N2pcs, with better search performance, and substantially larger and earlier target N2pcs in color as compared to shape search, indicating that finding color targets was overall easier than finding shape targets.

## 3 Experiment 2

The observation of Experiment 1 that color‐based search was generally easier and faster than shape‐based search might pose a problem when these two target dimensions are combined in a conjunction search task. If search guidance by color is more effective, a plausible search strategy might be to first use the color template to select all color‐matching objects in the visual field and then use the shape template to simply verify their target status (see Lee et al. 2018, for N2pc evidence for such a strategy during color/shape conjunction search). One way to counteract the choice of such a strategy is to increase the difficulty of color‐based search by increasing the difficulty of discriminating target and distractor colors (see Eimer and Grubert 2014, and Jenkins et al. 2017, for evidence that both color and shape guide attention when colored outline shapes rather than color‐filled objects are employed). In Experiment 2, we therefore employed more similar colors than in Experiment 1 and only presented outline shapes, to make the color task harder. In addition, we reduced the number of possible shapes, to make the shape task easier.

In addition to the two single feature conditions (color and shape), we also tested a conjunction task in Experiment 2 in which targets were defined by combinations of color and shape, and each search display also included a distractor object that either matched the target color or its shape. Each task had two load conditions, as in Experiment 1, and the critical comparisons were between the behavioral end electrophysiological load effects observed during the single feature and conjunction search tasks. If templates contain integrated object representations, these effects should be very similar across these two types of tasks. Alternatively, if attentional templates are always feature‐based, load effects should be much more pronounced in the conjunction search task, and possibly be twice as large, reflecting a load increase from 1 to 2 in the feature tasks, and from 2 to 4 in the conjunction task. A similar pattern should also be found for CDA components triggered in response to the target cues at the beginning of each trial. CDA amplitudes should generally be increased in high‐load as compared to low‐load trials, reflecting the parallel activation of two as compared one attentional template, respectively (e.g., Carlisle et al. 2011; Grubert et al. 2016). If attentional templates hold integrated representations of target features in the conjunction search task, load effects on the CDA should be very similar in all tasks. If they are always feature‐based, these effects should be much larger in the conjunction search task. Finally, this hypothesis can also be tested by examining whether partially target‐matching distractors attract attention and thus trigger N2pc components during conjunction search (e.g., Berggren and Eimer 2018; Lee et al. 2018), and this was also done in Experiment 2.

### 3.1 Methods

#### 3.1.1 Participants

Fifteen new participants aged 20–40 years (*M* = 25.1, SD = 5.0) were paid (£10/h) to participate in Experiment 2. Thirteen were female and two were male. All participants were right‐handed and had normal or corrected‐to‐normal vision without color deficiency (tested with the Ishihara color vision test; Ishihara 1918).

#### 3.1.2 Stimuli and Procedures

**FIGURE 4:**
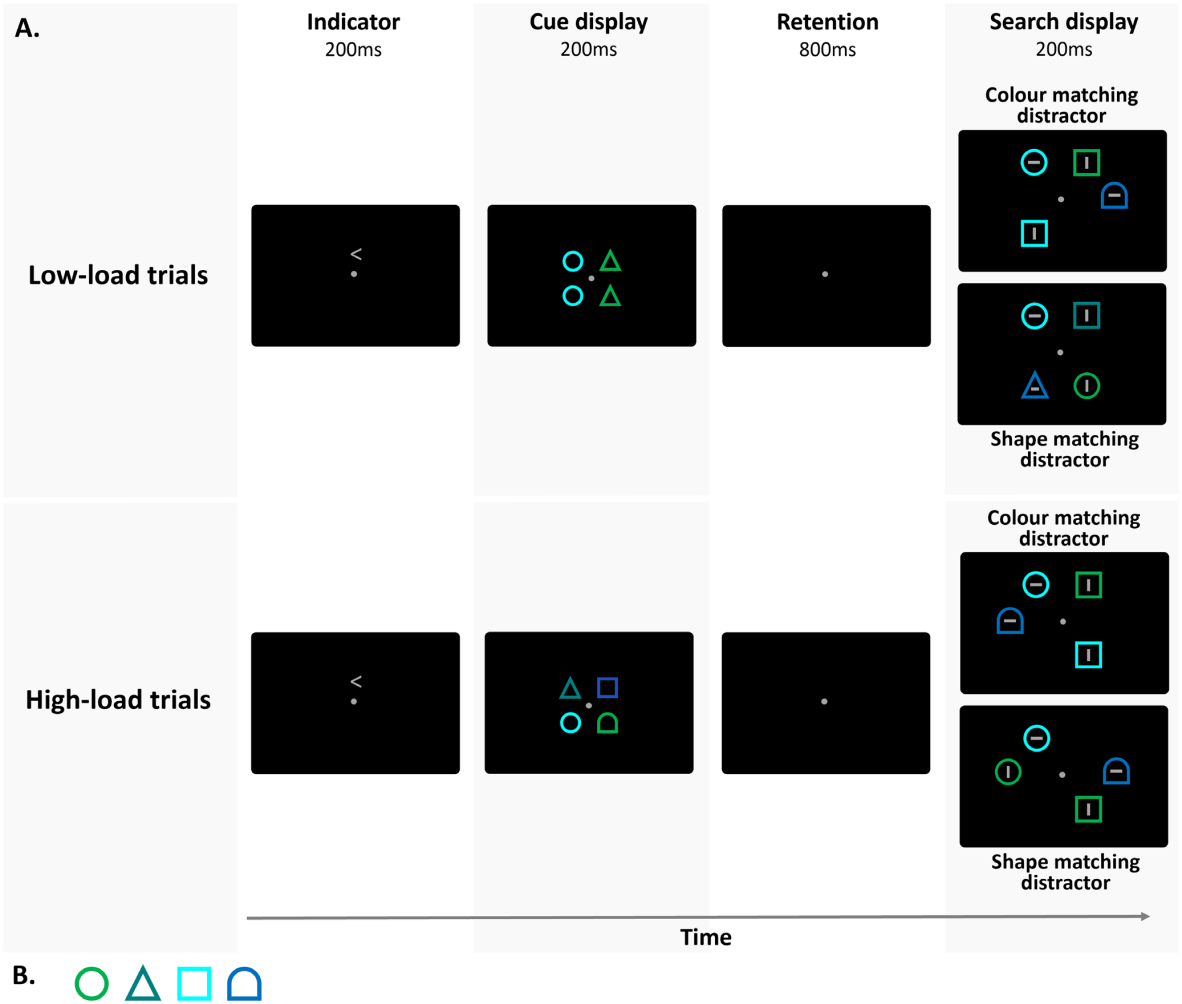
Schematic illustration of the stimuli and temporal trial sequence in Experiment 2. The trial sequence and timing were identical to Experiment 1, but cue displays as well as search displays were now physically identical in all task conditions, that is, color, shape, and conjunction tasks. Participants were instructed to search for the cued color(s), shape(s), or color and shape combination(s), respectively.

The stimuli and procedural aspects were identical to Experiment 1 with the few exceptions listed below. In addition to the *color* and *shape tasks* tested in Experiment 1, Experiment 2 contained a third blocked task condition, a *conjunction task*, in which participants searched for targets with a specific color and shape combination (Figure 4A).

To equate the neuronal responses to color and shape targets, we reduced task difficulty in the shape task by using four, rather than six possible target shapes (circle, triangle, square, and gate; 0.5° × 0.5°; Figure 4B). We also used four rather than six colors but made them perceptually more similar, to increase task difficulty in the color task (green: 0.621/0.335, turquoise: 0.285/0.616, cyan: 0.180/0.176, and blue: 0.462/0.465; equiluminant at ~9.5 cd/m^2^). In addition, all stimuli in the cue and search displays were now outline rather than filled shapes to reduce the amount of color information available during both template preparation and attentional guidance.

As in Experiment 1, search displays contained one target and three nontargets, presented at four out of the six possible lateralised stimulus locations as described in Experiment 1 (two in each hemifield at 1° from fixation). In each block of the color, shape, and conjunction task, each of the four colors, four shapes, and 16 color‐shape combinations, respectively, appeared equally often as the target. To ensure that conjunction targets could not solely be found based on the color or shape information alone, one of the nontargets in the conjunction task either matched the target color (50% of the trials) or the target shape (50%). In half of all trials, this partially matching distractor was presented on the same side as the target, in the other half, it was presented in the opposite hemifield. To keep the search displays physically identical between the three task conditions, the same logic was applied in the color and shape tasks. In the color task, one of the nontargets matched the shape of the color target, even though shape was completely task irrelevant, and vice versa, one of the nontargets matched the target color in the shape task, even though color was completely task irrelevant. The other non‐matching feature of the partially matching distractors, and the colors and shapes for the remaining two nontargets in each search display, were randomly chosen (without replacement) from the set of colors and shapes that were not used for the target. The response‐relevant lines within the search objects were gray in Experiment 2 (0.317/0.359; ~9.5 cd/m^2^).

In contrast to Experiment 1, the cue displays were now physically identical in all three task conditions and contained smaller versions of the search items (0.4° × 0.4°) that were also presented closer to fixation (±0.3° from both the horizontal and vertical axes) to prevent location overlap with the targets. *Low‐* and *high‐load* memory conditions were equiprobable and were presented in an intermixed fashion in each block. In low‐load trials, the two target cues on the relevant side were shown in one target color/shape/color & shape combination, while they were shown in two different target colors/shapes/color & shape combinations in high‐load trials. The two target cues in high‐load trials always differed with respect to both the target color and shape. As in Experiment 1, there was only ever one target in each search display and the colors and shapes of the irrelevant target cue never appeared in the search display. The color(s) and shapes(s) of the two cues on the irrelevant side of the cue display were randomly chosen (without replacement) from the set of colors and shapes that were not used for the target cues on the relevant cue side.

Experiment 2 was tested in two separate sessions because of the large number of trials required. The two sessions were completed by the same participants on two non‐consecutive days. Each session consisted of five consecutive blocks for each of the three tasks. Task order was counterbalanced between participants so that each of the three tasks was presented equally often first, second, and last across all participants. The task order of the first session was mirror‐reversed for each participant in the second session. All blocks contained eight trials for each combination of memory load (low‐, high‐load), cue side (left, right hemifield) and target side (left, right hemifield), for 64 trials per block and 1920 trials in total in Experiment 2.

#### 3.1.3 EEG Recording and Data Analyzes

EEG procedures were identical to Experiment 1. Trials with EEG artifacts, anticipatory, slow, missing, and error responses were excluded from analyzes. For the N2pc analysis, locked to the onset of the search displays, artifact rejection resulted in an exclusion of 16.7% of all trials in the color task (SD = 14.1%; ranging between 1.4% and 36.4% across participants), 13.9% of all trials in the shape task (SD = 11.9%; ranging between 0.2% and 33.1% across participants), and 16.3% in the conjunction task (SD = 13.0%; ranging between 0.7% and 36.7% across participants). EEG on the remaining trials was segmented into 500 ms epochs, from −100 to 400 ms relative to the onset of the search display. As in Experiment 1, N2pc mean amplitudes to targets in the search display were measured in the 190–290 ms time window after onset of the search displays. The threshold criterion for N2pc onset latencies was −0.7 μV in Experiment 2 (50% of the peak amplitude of the averaged target N2pc, pooled across all targets in all conditions of Experiment 2). Fractional peak latencies were defined as the time point at which the ascending flank of the jack‐knifed waveform reached 50% of its peak amplitude. In addition to the N2pc, in Experiment 2, we measured the contralateral delay activity (CDA) over PO7/8 in the 400–900 ms time widow after cue onset. The same artifact rejection criteria applied as for the N2pc analysis. For the CDA analysis, locked to the onset of the cue displays, artifact rejection resulted in an exclusion of 19.9% of all trials in the color task (SD = 15.6%; ranging between 1.3% and 39.3% across participants), 18.0% of all trials in the shape task (SD = 14.3%; ranging between 1.6% and 38.3% across participants), and 20.1% in the conjunction task (SD = 13.8%; ranging between 1.5% and 41.9% across participants).

### 3.2 Results

#### 3.2.1 Behavioral Results

Trials with anticipatory (< 200 ms) and exceedingly slow (> 1500 ms) responses were excluded from analysis (1.6% of all trials). BI scores^2^ in Experiment 2 (Figure 2, right panel) were subjected to a repeated‐measures ANOVA with the factors task (color, shape, conjunction) and memory load (low‐, high‐load), which revealed a main effect of load, *F*(1,14) = 321.2, *p* < 0.001, *η* ^2^_*p*_ = 0.96, *BF*_*incl*_ > 100, as participants were generally more efficient during low‐ (1.12) as compared to high‐load search (−1.12). There was no main effect of task, *F*(2,28) = 3.9, *p* = 0.064 *η* ^2^_*p*_ = 0.22, *BF*_*incl*_ = 2.5, but task interacted with load, *F*(2,28) = 7.6, *p* = 0.009, *η* ^2^_*p*_ = 0.35, *BF*_*incl*_ = 14.4, because load effects (difference between low‐ and high‐load BI scores) were significantly increased in the conjunction task (2.60), both in comparison with the color task (1.89), *t*(14) = 4.5, *p* < 0.001, *d* = 0.42, *BF*_*10*_ = 44.4, and the shape task (2.25), *t*(14) = 2.3, *p* = 0.036, *d* = 0.60, *BF*_*10*_ = 4.4. However, load effects on BI scores were comparable in the color and shape tasks, *t*(14) = 1.5, *p* = 0.175, *d* = 0.42, *BF*_*10*_ = 0.7. Because there were no reliable task and load effect differences between the color and shape tasks, the two single feature tasks were pooled for the following ERP analyzes.

#### 3.2.2 N2pc Results

**FIGURE 5:**
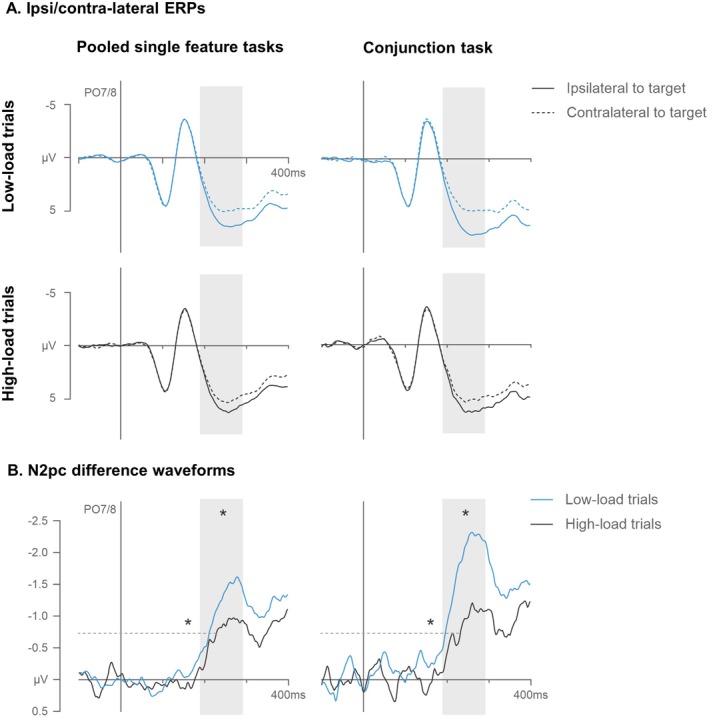
Grand‐averaged ERPs measured at electrodes PO7/8 contralateral and ipsilateral to the targets in low‐ and high‐load trials of the single feature tasks (pooled for color and shape) and the conjunction tasks in Experiment 2 (A). The respective difference waves (contra‐ minus ipsilateral ERPs) are shown below (B). The shaded areas mark the N2pc time window (190–290 ms after search display onset) and the dashed lines indicate the onset latency criterion (−0.7 μV). Asterisks mark statistically reliable load effects (differences between high‐ and low‐load trials) measured in N2pc mean amplitudes (asterisks above amplitudes) and onset latencies (asterisks above onset criterion).

Figure 5A shows grand‐averaged ERPs in response to the targets in the low‐ and high‐load trials of the single feature (pooled for color and shape^3^) and feature conjunction tasks, together with the corresponding N2pc difference waves (Figure 5B). While the single feature N2pcs mirrored the N2pcs measured in Experiment 1, with slightly attenuated and delayed N2pcs in high‐ as compared to low‐load trials, this load effect seemed to be substantially increased in the conjunction task. To verify these observations, N2pc mean amplitudes were fed into a repeated‐measure ANOVA with the factors task (single‐feature, conjunction), memory load (low‐, high‐load), and laterality (electrode contralateral, ipsilateral to the target). The ANOVA uncovered a main effect of laterality, *F*(1,14) = 64.9, *p* < 0.001, *η* ^2^_*p*_ = 0.82, *BF*_*incl*_ > 100, which interacted with task, *F*(1,14) = 33.7, *p* < 0.001, *η* ^2^_*p*_ = 0.71, *BF*_*incl*_ > 100, and load, *F*(1,14) = 74.4, *p* < 0.001, *η* ^2^_*p*_ = 0.84, *BF*_*incl*_ > 100. In other words, N2pcs were generally larger when they were triggered in response to conjunction (−1.3 μV) rather than single‐feature targets (−0.9 μV), and in low‐load (−1.5 μV) as compared to high‐load trials (−0.8 μV). Critically, the ANOVA also revealed a significant three‐way interaction, *F*(1,14) = 5.3, *p* = 0.022, *η* ^2^_*p*_ = 0.32, *BF*_*incl*_ = 62.4, because the load effects (low‐ minus high‐load amplitudes) were substantially increased in the conjunction (−1.0 μV) as compared to the single‐feature tasks (−0.4 μV).

N2pc onset latencies measured with a fixed onset criterion at −0.7 μV were fed into an ANOVA with the factors task (single‐feature, conjunction) and memory load (low‐, high‐load). There was no main effect of task, *F*_*c*_(1,14) = 1.1, *p* = 0.213, *η* ^2^_*pc*_ = 0.07, *BF*_*incl*_ = 0.5, but a significant main effect of load, *F*_*c*_(1,14) = 42.5, *p* < 0.001, *η* ^2^_*pc*_ = 0.75, *BF*_*incl*_ > 100, and a reliable interaction, *F*_*c*_(1,14) = 4.1, *p* = 0.028, *η* ^2^_*pc*_ = 0.23, *BF*_*incl*_ = 2.7 (marginal at the Bayesian level). N2pc onset latencies were generally delayed in high‐load (229 ms) as compared to low‐load trials (203 ms), but this load effect (high‐ minus low‐load latencies), was substantially increased in the conjunction (35 ms) as compared to the single feature tasks (18 ms). The same results were found for fractional (50%) peak latencies: There was no main effect of task, *F*_*c*_(1,14) = 1.3, *p* = 0.274, *η* ^2^_*pc*_ = 0.08, *BF*_*incl*_ = 0.5, but a main effect of load, *F*_*c*_(1,14) = 9.01, *p* = 0.010, *η* ^2^_*pc*_ = 0.39, *BF*_*incl*_ = 2.7 (marginal at the Bayesian level), and a reliable interaction, *F*_*c*_(1,14) = 8.5, *p* = 0.011, *η* ^2^_*pc*_ = 0.38, *BF*_*incl*_ = 36.5, indicating that load effects were more pronounced in the conjunction (25 ms) than the single feature task (1 ms).

##### 3.2.2.1 Effects of Partially Matching Distractors on Target N2pcs

**FIGURE 6:**
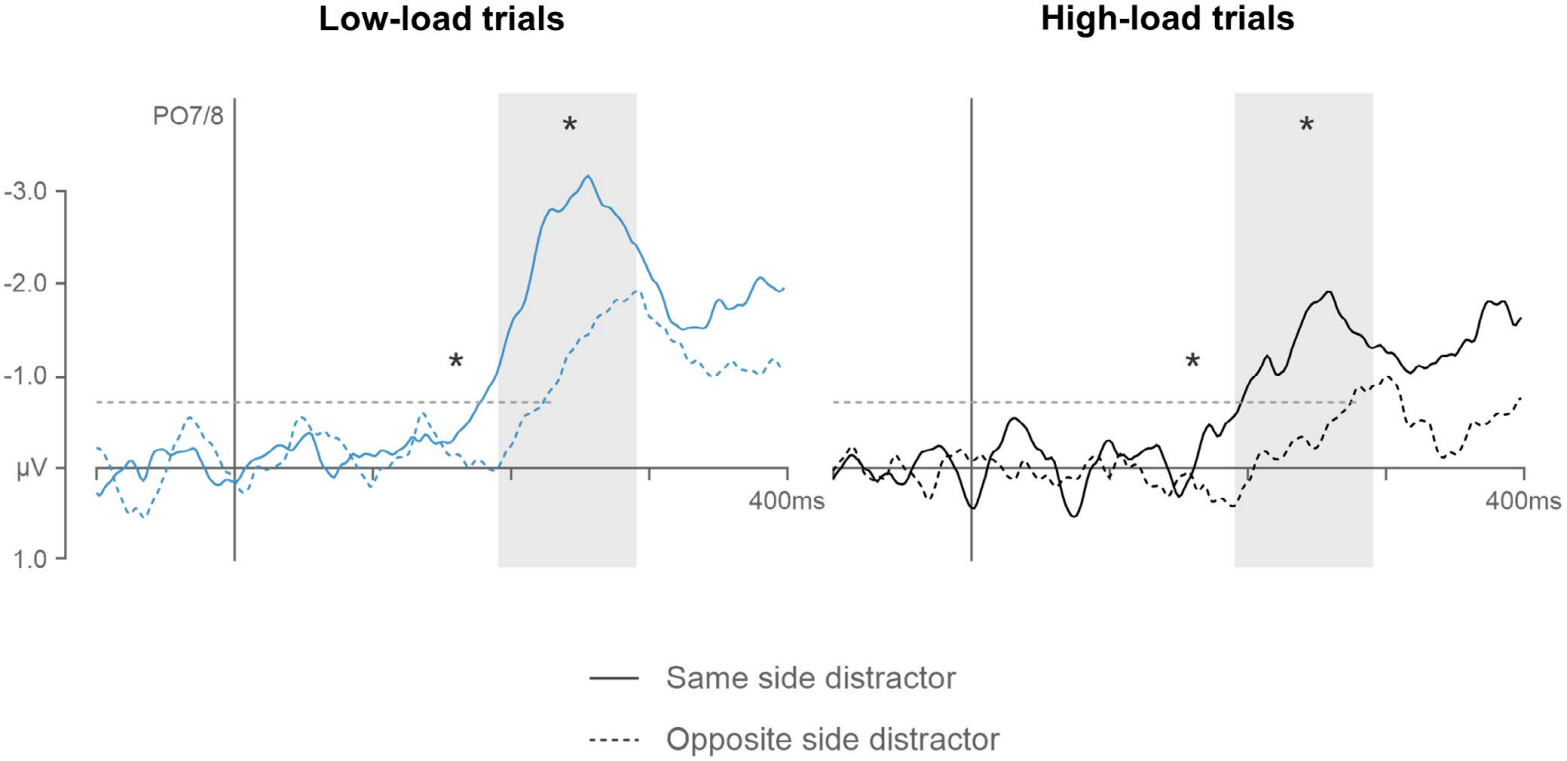
N2pc difference waves in low‐ and high‐load trials of the conjunction task in Experiment 2, separately for trials in which partially matching distractors were presented in the same or opposite hemifield of the target. N2pcs are pooled for distractors that matched the target color or shape, respectively (separate N2pcs for color and shape matching distractors are shown in Figure S2). The shaded areas mark the N2pc time window (190–290 ms after search display onset) and the dashed lines indicate the onset latency criterion (−0.7 μV). Asterisks mark statistically reliable amplitude (asterisks above amplitudes) and latency differences (asterisks above onset criterion) between N2pcs triggered in response to targets with same or opposite side distractors.

If templates are feature‐based during conjunction search, distractors that share one of the target‐defining features should attract attention because they match one of the feature‐based attentional templates. We tested this prediction by measuring the effects of color‐ and shape‐matching distractors on target N2pcs in the conjunction search task. Figure 6 shows N2pc difference waves in low‐ and high‐load trials with a partially matching distractor in the same or opposite hemifield relative to the target (separate N2pcs for color and shape matching distractors, respectively, are shown in Figure S2). N2pc mean amplitudes in these trials were subjected to a repeated‐measure ANOVA with the factors laterality, memory load, distractor type (color, shape matching), and distractor side (same, opposite side to target). An interaction between laterality and distractor side, *F*(1,14) = 64.5, *p* < 0.001, *η* ^2^_*p*_ = 0.82, *BF*_*incl*_ > 100, showed that N2pc mean amplitudes were attenuated when a partially matching distractor was presented in the opposite (−0.7 μV) as compared to the same side of the target (−1.9 μV). This reduction of target N2pc amplitudes by opposite‐side distractors was virtually identical for color and shape matching distractors (−1.2 μV), *F*(1,14) < 1, *p* = 0.946, *η* ^2^_*p*_ < 0.01, *BF*_*incl*_ = 0.4, and was also comparable in low‐load (−1.4 μV) and high‐load trials (−1.1 μV), *F*(1,14) = 2.4, *p* = 0.145, *η* ^2^_*p*_ = 0.146, *BF*_*incl*_ = 0.9. The same pattern was found for N2pc onset latencies fixed at −0.7 μV. A repeated‐measure ANOVA with the factors memory load, distractor type, and distractor side produced a main effect of distractor side, *F*_*c*_(1,14) = 10.6, *p* = 0.002, *η* ^2^_*pc*_ = 0.43, *BF*_*incl*_ = 7.9, as target N2pcs emerged later when the partially matching distractor was on the opposite side (244 ms) versus in the same hemifield than the target (185 ms). This distractor‐induced N2pc onset delay was comparable for color matching (72 ms) and shape matching (46 ms) distractors, *F*_*c*_(1,14) = 0.5, *p* = 0.408, *η* ^2^_*pc*_ = 0.04, *BF*_*incl*_ = 0.3, and in low‐load (43 ms) and high‐load trials (75 ms), *F*_*c*_(1,14) = 1.0, *p* = 0.229, *η* ^2^_*pc*_ = 0.07, *BF*_*incl*_ = 0.9. Almost the same results were found for fractional (50%) peak latencies: There was a main effect of distractor side, *F*_*c*_(1,14) = 35.4, *p* < 0.001, *η* ^2^_*pc*_ = 0.72, *BF*_*incl*_ > 100, with distractor effects being comparable for color (63 ms) and shape distractors (41 ms), *F*_*c*_(1,14) = 2.2, *p* = 0.159, *η* ^2^_*pc*_ = 0.14, *BF*_*incl*_ = 0.4. However, distractor effects were now more pronounced in the high‐load (71 ms) than the low‐load condition (32 ms) condition, *F*_*c*_(1,14) = 6.3, *p* = 0.025, *η* ^2^_*pc*_ = 0.31, *BF*_*incl*_ = 2.8 (marginal at the Bayesian level).

#### 3.2.3 CDA Results

**FIGURE 7:**
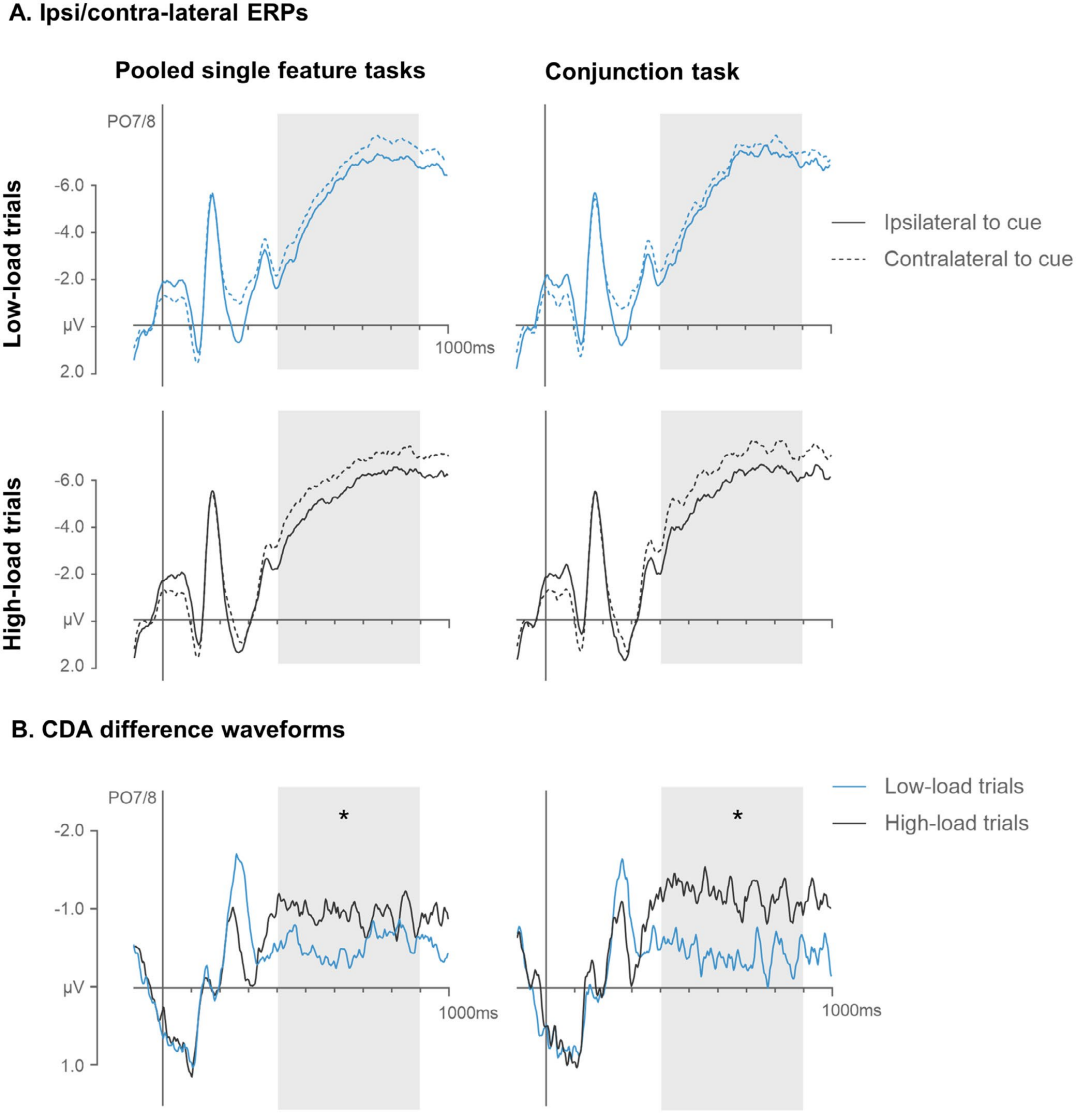
Grand‐averaged ERPs measured at electrodes PO7/8 contralateral and ipsilateral to the target cues in low‐ and high‐load trials of the single feature tasks (pooled for color and shape) and the conjunction tasks in Experiment 2 (A). The respective difference waves (contra‐ minus ipsilateral ERPs) are shown below (B). The shaded areas mark the CDA time windows (400–900 ms after cue display onset). Asterisks mark statistically reliable load effects (differences between high‐ and low‐load trials) measured in CDA mean amplitudes.

Figure 7A shows grand‐averaged ERPs at electrode sites PO7/8 contralateral and ipsilateral to the target cues in low‐ and high‐load trials of the single feature and feature conjunction tasks. CDA components were elicited in the 400–900 ms time interval after cue display onset in all four conditions. The corresponding CDA difference waves are shown in Figure 7B. CDAs were generally increased in high‐load as compared to low‐load trials, but this load effect seemed to be exaggerated in the conjunction as compared to the single feature tasks. This was substantiated by means of a repeated‐measure ANOVA with the factors task (single feature, conjunction), memory load (low, high‐load), and laterality (electrode contralateral, ipsilateral to the target cues). A main effect of laterality, *F*(1,14) = 11.7, *p* = 0.004, *η* ^2^_*p*_ = 0.46, was accompanied by a significant Laterality × Load interaction, *F*(1,14) = 11.5, *p* = 0.004, *η* ^2^_*p*_ = 0.45, and a significant three‐way interaction, *F*(1,14) = 5.0, *p* = 0.042, *η* ^2^_*p*_ = 0.26. CDA mean amplitudes were generally larger in high‐load (−0.9 μV) than low‐load trials (−0.6 μV), but these load effects (high minus low‐load amplitudes) were substantially increased in the conjunction (−0.6 μV) as compared to the single feature tasks (−0.3 μV). These findings were reinforced by a complementary Bayesian ANOVA, which provided support for the main effect of laterality (*BF*_*incl*_ = 39.4), the Laterality × Load interaction (*BF*_*incl*_ = 23.8), and the three‐way interaction (*BF*_*incl*_ = 7.5).

### 3.3 Discussion of Experiment 2

The results from the single‐feature search tasks confirmed the results of Experiment 1. Search performance was worse in high‐load as compared to low‐load trials, and these load effects were comparable between the color and shape tasks. N2pc amplitudes and latencies were attenuated and delayed in high‐load as compared to low‐load trials. In fact, amplitude load effects measured in the (pooled) single feature conditions of Experiment 1 and 2 were the same (−0.4 μV), and the latency effects were almost identical (15 vs. 18 ms in Experiments 1 and 2, respectively), and suggest the presence of competitive interactions between two simultaneously active feature templates in high‐load trials (see also Grubert and Eimer 2023; Grubert et al. 2025; Ort et al. 2019). One notable difference to Experiment 1 was that search performance was no longer better with color‐defined as compared to shape‐defined targets. BI scores were now comparable between the color and shape tasks, and this was explicitly tested in a follow‐up *t*‐test, *t*(14) = 1.6, *p* = 0.283, *d* = 0.40, *BF10* < 1, demonstrating that using colored outline shapes and increasing color similarity eliminated the general advantage for color observed in Experiment 1.

The critical question addressed in Experiment 2 was whether the behavioral and electrophysiological effects of load would be systematically larger in the conjunction search task relative to the two feature tasks. This was clearly the case. Load effects on search performance were substantially more pronounced in the conjunction task, indicating that increasing the number of possible targets affected search efficiency much more strongly than in the feature tasks. An analogous pattern was found for target N2pcs. Again, memory load effects on N2pc amplitudes and onset latencies were substantially larger in the conjunction task (−1.0 μV; 35 ms) as compared to the single feature tasks (–0.4 μV; 18 ms), indicating that increasing the number of possible targets affected attentional guidance more strongly during conjunction search.

These results are fully in line with the hypothesis that conjunction search was guided by individual feature templates. While there were one versus two templates active during low‐ and high‐load single feature search, in the conjunction task participants had to activate two versus four templates concurrently in low versus high load trials, resulting in more interference between templates and less efficient guidance of search (see Kerzel and Grubert 2022, for model predictions of increased costs for larger numbers of attentional templates that are activated in parallel). This was further confirmed by the analysis of target N2pcs as a function of the location of the partially matching distractors. Target N2pcs were substantially attenuated and delayed when the target and the partially matching distractor were in opposite as compared to the same hemifield. This pattern indicates that distractors which either matched the target color or shape captured attention and thus triggered an N2pc, which combined with the target N2pc when both items appeared on the same side but eliminated the early phase of the target N2pc when they appeared in different hemifields and therefore triggered N2pcs of opposite polarity. This ability of partially matching distractors to capture attention in the conjunction task provides additional strong evidence that attentional guidance was feature‐based.

Finally, the CDA results obtained during the interval between the cue and search displays in Experiment 2 provide further evidence for the activation of feature‐based attentional templates during conjunction search. Load effects in the single feature tasks mirrored previous findings of increased CDA components when the number of possible targets in the upcoming search display is increased (e.g., Carlisle et al. 2011; Grubert et al. 2016). Critically, these load effects were significantly larger in the conjunction than the single feature tasks (−0.6 μV vs. −0.3 μV), demonstrating that increasing load increased the number of working memory representations maintained during search preparation more strongly in the conjunction task, and likely doubled this number. Had the attentional templates activated in the conjunction task represented targets in an integrated object‐based fashion, no such differential CDA load effects should have been observed between feature and conjunction search tasks (e.g., Luck and Vogel 1997; Vogel et al. 2001). The fact that such differences were clearly present is therefore in line with the hypothesis that attentional templates for individual target features were activated independently in the conjunction task.

It is important to note that our CDA analyzes focused on differential load effects between tasks rather than absolute CDA amplitudes because previous findings showed that CDA amplitudes are sensitive not only to the number of features but also to the number of stimulus locations occupied by these features (e.g., Balaban and Luria 2016; Luria and Vogel 2011; Rabbitt et al. 2017). By comparing net load effects (i.e., the difference in CDA amplitude between high and low load conditions), we avoided this confound, as these differences always reflect the contrast between activity at two versus one location in both the feature and conjunction tasks. Another potential problem for the interpretation of these CDA effects is that CDA amplitudes are known to be sensitive to the complexity of memorized items (e.g., colors versus random polygon shapes; Gao et al. 2009). Since targets in the conjunction task were arguably more complex than in the feature tasks, differential CDA load effects could in principle reflect the difference between storing one and two complex versus simpler items. This possibility is unlikely, as Luria and Vogel (2011, Exp.1) have shown that manipulating complexity by increasing the number of relevant features of a single memorized object (e.g., color only versus color and shape, as in the present experiment) does not affect CDA amplitudes (see Luria et al. 2016, for a detailed discussion of the links between CDA components and stimulus complexity).

## 4 General Discussion

Previous research has shown that attentional templates used for guiding attention toward task‐relevant locations in visual search can be set up during the preparation for upcoming search episodes (see Eimer 2014, for a review). When observers search for more than one possible target feature, multiple preparatory attentional templates can be activated concurrently, although this comes at a cost relative to single‐feature search (see Ort and Olivers 2020, for a review). Here, we investigated the nature of attentional templates that are used in conjunction search tasks where target objects are defined by a combination of features from different dimension. The question was whether all these features are represented independently in feature‐specific templates, or whether such templates can store target information in an integrated object‐based fashion.

The results of this study provide unequivocal evidence that both feature and conjunction search is guided by feature‐based attentional templates. In Experiment 1, we obtained behavioral and electrophysiological evidence that two feature‐based templates are activated in parallel when the number of possible search targets is increased from one to two, and that this increase in template load produces costs both at the level of search performance as well as at the level of electrophysiological markers of attentional guidance toward target objects (N2pc components). Importantly, this was shown not only for search tasks with color‐defined targets, as in previous research, but also when observers searched for targets defined by shape.

In Experiment 2, we contrasted the template load effects observed during feature search with the effects that emerge when targets are defined by a conjunction of color and shape. Critically, the behavioral and electrophysiological effects of increasing the number of possible targets to two were much more pronounced during conjunction as compared to feature search. This was the case for search performance, the amplitudes and latencies of target N2pc components, as well as for CDA components measured during the interval before the presentation of the search display. The fact that these memory load effects were substantially increased (and essentially twice as large) in the conjunction task relative to the feature tasks suggests that twice the amount of target‐related information was activated during search preparation when targets were defined by a feature conjunction. This is of course entirely in line with the hypothesis that this information is represented in a feature‐based fashion, and not as integrated object‐based attentional templates. Further evidence for this conclusion comes from the finding that the location of distractors which matched either the target color or shape affected the target N2pc components. N2pcs were attenuated and delayed when partially matching distractors appeared in the opposite side relative to the target as compared to same‐side trials. These differences are most likely due to the fact that these partially matching distractors captured attention and thus elicited N2pc components, which were of opposite polarity relative to target N2pcs on opposite‐side trials. The ability of partially matching distractors to trigger N2pcs indicative of attentional capture even though the search target was present in the same display provides additional strong evidence that distinct attentional templates for target color and shape were activated in parallel and independently during conjunction search.

Overall, these observations are in accordance with models of visual search that assume that the guidance of attention by preparatory attentional templates is always feature‐based (e.g., Bundesen 1990; Huang and Pashler 2007; Itti and Koch 2001; Müller and Krummenacher 2006; Wolfe 2021), even when target objects are defined by feature conjunctions. Numerous previous behavioral and electrophysiological studies have also supported this assumption (e.g., Becker et al. 2020; Berggren and Eimer 2018; Dent 2023; Eimer and Grubert 2014; Jenkins et al. 2017; Lee et al. 2018; Rutishauser and Koch 2007; Williams and Reingold 2001). However, because the present study employed the N2pc as a marker of template‐based attentional guidance, this conclusion depends to a large degree on whether this interpretation of the N2pc is correct. If this component primarily reflected processes that take place after the initial selection of target objects, such as target/nontarget discrimination, the effects of template load on N2pc amplitudes observed in Experiment 2 may reflect differences between feature and conjunction search that exclusively emerge at these later stages rather than during attentional guidance. However, several previous studies have shown that at least during its early stages, the N2pc does in fact reflect the allocation of attention to possible targets. For example, Mazza et al. (2007) compared target‐induced N2pc components in a task where color‐defined singleton targets either had to be simply localized and a task where their shapes had to be discriminated. N2pcs were identical in both tasks, whereas a subsequent sustained contralateral negativity was only observed in the discrimination task. This suggests that the N2pc primarily reflects spatial selection rather than later object identification and discrimination processes. Analogous observations come from conjunction search tasks (e.g., Eimer and Grubert 2014; Jenkins et al. 2017; Berggren and Eimer 2018). As mentioned earlier, these studies consistently found that the N2pc to conjunctively defined targets was initially identical in size to the sum of N2pcs by partially target‐matching distractors and only became larger beyond 250 ms after search display onset. This pattern strongly suggests that up to this point in time, the N2pc is solely sensitive to feature‐based attentional guidance, and not yet to target/nontarget discrimination processes. In this context, it is important to note that the differential effects of template load on N2pc amplitudes during feature versus conjunction search emerged already from about 200 ms post‐stimulus (see Figure 5). This seems to provide clear evidence that these effects were indeed generated during the template‐guided allocation of attention to target objects.

It is generally believed that attentional templates are held in working memory, and it is also clear that working memory representations can be object‐based (e.g., Balaban and Luria 2016; Cowan 2001; Delvenne and Bruyer 2004; Olson and Jiang 2002; Luck and Vogel 1997; Quinlan and Cohen 2011; Vogel et al. 2001). So, why should it be the case that attentional templates are always feature‐based even when object‐based representations are in principle available, and would result in a reduction in overall memory load, particularly during conjunction search tasks with multiple possible target objects? It is plausible to assume that attentional templates are feature‐based rather than object‐based because this is helpful for their role of guiding attention toward possible target locations in visual search. For example, in many search tasks where the similarity between targets and distractor objects is high, attentional templates must be highly precise to discriminate a target from its surrounding distractors (e.g., Kerzel and Cong 2021; Yu et al. 2023). Evidence from working memory research suggests that integrated object representations are less precise than feature‐based representations (e.g., Fougnie et al. 2010), and this may limit their ability to guide attention in difficult search tasks.

However, there may be another more fundamental reason why attentional templates are feature based. To control spatial attention adaptively, these templates must access the information that is available after an initial rapid and presumably parallel extraction of information from across the entire visual field. Such pre‐attentive maps of current visual input are likely to contain primarily information about the presence of specific features at specific locations. Even though such maps may contain some additional structure beyond elementary feature signals (e.g., Wolfe and Bennett 1997), it is very unlikely that they already include object‐based information resulting from the integration of features into object files. If this was the case, object‐based attentional templates and pre‐attentive visual scanning mechanisms would not be able to interact usefully, because their incommensurate representational format. In contrast, attentional templates that are purely feature‐based should be able to access pre‐attentive feature information directly and use this information to guide attention to potentially task‐relevant locations in visual search displays. It is interesting to note that this constraint does not apply to another putative function of attentional templates, namely, their role in the recognition of attended objects in a search display as targets or nontargets. Because such template matching processes takes place after attentional guidance is complete, they may well involve object‐based representations. It would be interesting for future research to investigate whether such a dissociation between feature‐based templates for guidance and object‐based templates for recognition does in fact exist.

## Author Contributions

**Ziyi Wang:** investigation, writing – original draft, visualization, writing – review and editing, formal analysis, data curation, conceptualization, methodology. **Mikel Jimenez:** writing – original draft, writing – review and editing, investigation, methodology, conceptualization. **Martin Eimer:** conceptualization, funding acquisition, writing – original draft, writing – review and editing. **Anna Grubert:** conceptualization, funding acquisition, writing – original draft, writing – review and editing, methodology, project administration, supervision.

## Conflicts of Interest

The authors declare no conflicts of interest.

## Supporting information


**Figure S1:** N2pc difference waves in low‐ and high‐load trials in the color and shape tasks of Experiment 2. The shaded areas mark the N2pc time window (190–290 ms after search display onset) and the dashed lines indicate the onset latency criterion (−0.7 μV). Asterisks mark statistically reliable load effects (differences between high‐ and low‐load trials) measured in N2pc mean amplitudes. Mean amplitudes were subjected to a repeated‐measures ANOVA with the factors task (color, shape), memory load (low‐, high‐load), and laterality (electrode contralateral, ipsilateral to the target). A main effect of laterality, *F*(1,14) = 57.1, *p* < 0.001, *η* ^2^_*p*_ = 0.80, *BF*_*incl*_ > 100, interacted with load, *F*(1,14) = 33.0, *p* < 0.001, *η* ^2^_*p*_ = 0.70, *BF*_*incl*_ > 100, revealing that significant N2pcs were triggered across task conditions but that these were larger in low‐load (−1.2 μV) compared to high‐load trials (−0.7 μV). However, there were no significant interactions involving the factor task, *F*(1,14) < 2.1, *p* > 0.167, *η* ^2^_*p*_ < 0.13, *BF*_*incl*_ < 1, demonstrating that N2pc mean amplitudes (−1.0 versus −0.9 μV, respectively) and load effects (low‐ minus high‐load amplitudes; −0.3 versus −0.5 μV, respectively) did not differ between the color and shape tasks. The equivalent ANOVA for onset latencies did not produce any significant effects at all, all *F*_*c*_(1,14) < 2.1, *p* > 0.094, *η* ^2^_*pc*_ < 0.13. N2pc onset latencies (213 versus 232 ms, respectively) and load effects (high‐ minus low‐load latencies; 20 versus 34 ms, respectively) were identical in the color and shape tasks of Experiment 2.

**Figure S2:** N2pc difference waves in low‐ and high‐load trials of the conjunction task in Experiment 2, separately for trials in which partially matching distractors were presented in the same or opposite hemifield of the target. N2pcs are shown separately for distractors that matched the target color or shape, respectively. The shaded areas mark the N2pc time window (190–290 ms after search display onset) and the dashed lines indicate the onset latency criterion (−0.7 μV). Asterisks mark statistically reliable amplitude (asterisks above amplitudes) and latency differences (asterisks above onset criterion) between N2pcs triggered in response to targets with same or opposite side distractors.

**Table S1:** Mean reaction times (RTs; in milliseconds) and error rates (ER; % of trials) measured in low‐ and high‐load trials of the color and shape tasks in Experiment 1. Brackets contain standard deviations.

**Table S2:** Mean reaction times (RTs; in milliseconds) and error rates (ER; % of trials) measured in low‐ and high‐load trials of the color, shape, and conjunction tasks in Experiment 2. Brackets contain standard deviations.

## Data Availability

The data that support the findings of this study are available from the corresponding author upon reasonable request.

## Endnotes

^1^
A large effect size was expected to replicate partial eta squared values (ηp2) of 0.55, which we measured in a previous N2pc experiment in which participants searched for one versus two target colors (Color load × Laterality interaction in Experiment 1 of Grubert et al. 2016, p. 1953). Note that the sample size of that reference study was also 15.

^2^
Separate mean RTs and error rates for Experiment 2 are presented in the Table S2.

^3^
Separate color and shape N2pc difference waves are shown in Figure S1. Statistics are given in the figure legend to prove that the stimulus changes in Experiment 2 helped to equate neuronal responses to color and shape targets and that color and shape N2pcs were statistically identical in Experiment 2.
