# Supplementary material for: The Guidance of Attentional Selectivity in Visual Search Is Always Feature‐Based: Behavioral and Electrophysiological Evidence From Feature and Conjunction Search Tasks
Source: Psychophysiology. 2025 Oct 22;62(10):e70169. doi: 10.1111/psyp.70169 (PMC12541687; doi:10.1111/psyp.70169)
Supplement: Supplementary file 1 — Figure S1: N2pc difference waves in low‐ and high‐load trials in the color and shape tasks of Experiment 2. The shaded areas mark the N2pc time window (190–290 ms after search display onset) and the dashed lines indicate the onset latency criterion (−0.7 μV). Asterisks mark statistically reliable load effects (differences between high‐ and low‐load trials) measured in N2pc mean amplitudes. Mean amplitudes were subjected to a repeated‐measures ANOVA with the factors task (color, shape), memory load (low‐, high‐load), and laterality (electrode contralateral, ipsilateral to the target). A main effect of laterality, F(1,14) = 57.1, p < 0.001, η 2 p = 0.80, BF incl > 100, interacted with load, F(1,14) = 33.0, p < 0.001, η 2 p = 0.70, BF incl > 100, revealing that significant N2pcs were triggered across task conditions but that these were larger in low‐load (−1.2 μV) compared to high‐load trials (−0.7 μV). However, there were no significant interactions involving the factor task, F(1,14) < 2.1, p > 0.167, η 2 p < 0.13, BF incl < 1, demonstrating that N2pc mean amplitudes (−1.0 versus −0.9 μV, respectively) and load effects (low‐ minus high‐load amplitudes; −0.3 versus −0.5 μV, respectively) did not differ between the color and shape tasks. The equivalent ANOVA for onset latencies did not produce any significant effects at all, all F c (1,14) < 2.1, p > 0.094, η 2 pc < 0.13. N2pc onset latencies (213 versus 232 ms, respectively) and load effects (high‐ minus low‐load latencies; 20 versus 34 ms, respectively) were identical in the color and shape tasks of Experiment 2. [file PSYP-62-e70169-s002.docx]

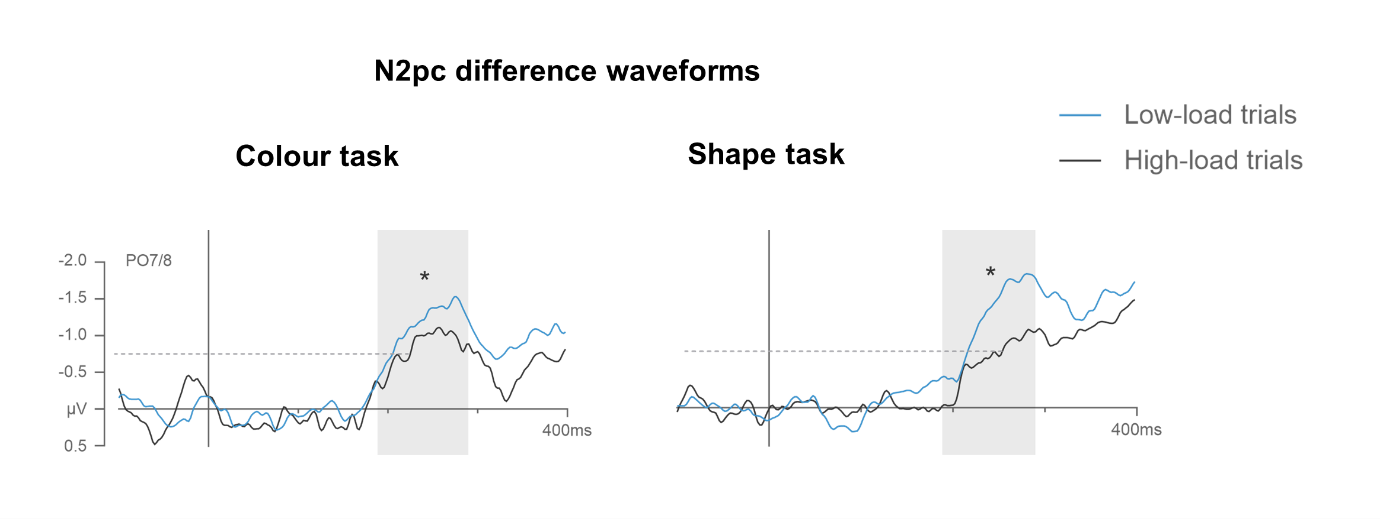


**Figure S1.** N2pc difference waves in low- and high-load trials in the colour and shape tasks of Experiment 2. The shaded areas mark the N2pc time window (190-290ms after search display onset) and the dashed lines indicate the onset latency criterion (-0.7μV). Asterisks mark statistically reliable load effects (differences between high- and low-load trials) measured in N2pc mean amplitudes. Mean amplitudes were subjected to a repeated-measures ANOVA with the factors task (colour, shape), memory load (low-, high-load), and laterality (electrode contralateral, ipsilateral to the target). A main effect of laterality, *F*(1,14)=57.1, *p*<.001, *η^2^_p_*=.80, *BF_incl_*>100, interacted with load, *F*(1,14)=33.0*, p*<.001, *η^2^_p_*=.70, *BF_incl_*>100, revealing that significant N2pcs were triggered across task conditions but that these were larger in low-load (-1.2μV) compared to high-load trials (-0.7μV). However, there were no significant interactions involving the factor task, *F*(1,14)<2.1, *p*>.167, *η^2^_p_*<.13, *BF_incl_*<1, demonstrating that N2pc mean amplitudes (-1.0μV versus -0.9μV, respectively) and load effects (low- minus high-load amplitudes; -0.3μV versus -0.5μV, respectively) did not differ between the colour and shape tasks. The equivalent ANOVA for onset latencies did not produce any significant effects at all, all *F_c_*(1,14)<2.1, *p*>.094, *η^2^_pc_*<.13. N2pc onset latencies (-213ms versus 232ms, respectively) and load effects (high- minus low-load latencies; 20ms versus 34ms, respectively) were identical in the colour and shape tasks of Experiment 2.
