# Supplementary material for: The Guidance of Attentional Selectivity in Visual Search Is Always Feature‐Based: Behavioral and Electrophysiological Evidence From Feature and Conjunction Search Tasks
Source: Psychophysiology. 2025 Oct 22;62(10):e70169. doi: 10.1111/psyp.70169 (PMC12541687; doi:10.1111/psyp.70169)
Supplement: Supplementary file 2 — Figure S2: N2pc difference waves in low‐ and high‐load trials of the conjunction task in Experiment 2, separately for trials in which partially matching distractors were presented in the same or opposite hemifield of the target. N2pcs are shown separately for distractors that matched the target color or shape, respectively. The shaded areas mark the N2pc time window (190–290 ms after search display onset) and the dashed lines indicate the onset latency criterion (−0.7 μV). Asterisks mark statistically reliable amplitude (asterisks above amplitudes) and latency differences (asterisks above onset criterion) between N2pcs triggered in response to targets with same or opposite side distractors. [file PSYP-62-e70169-s004.docx]

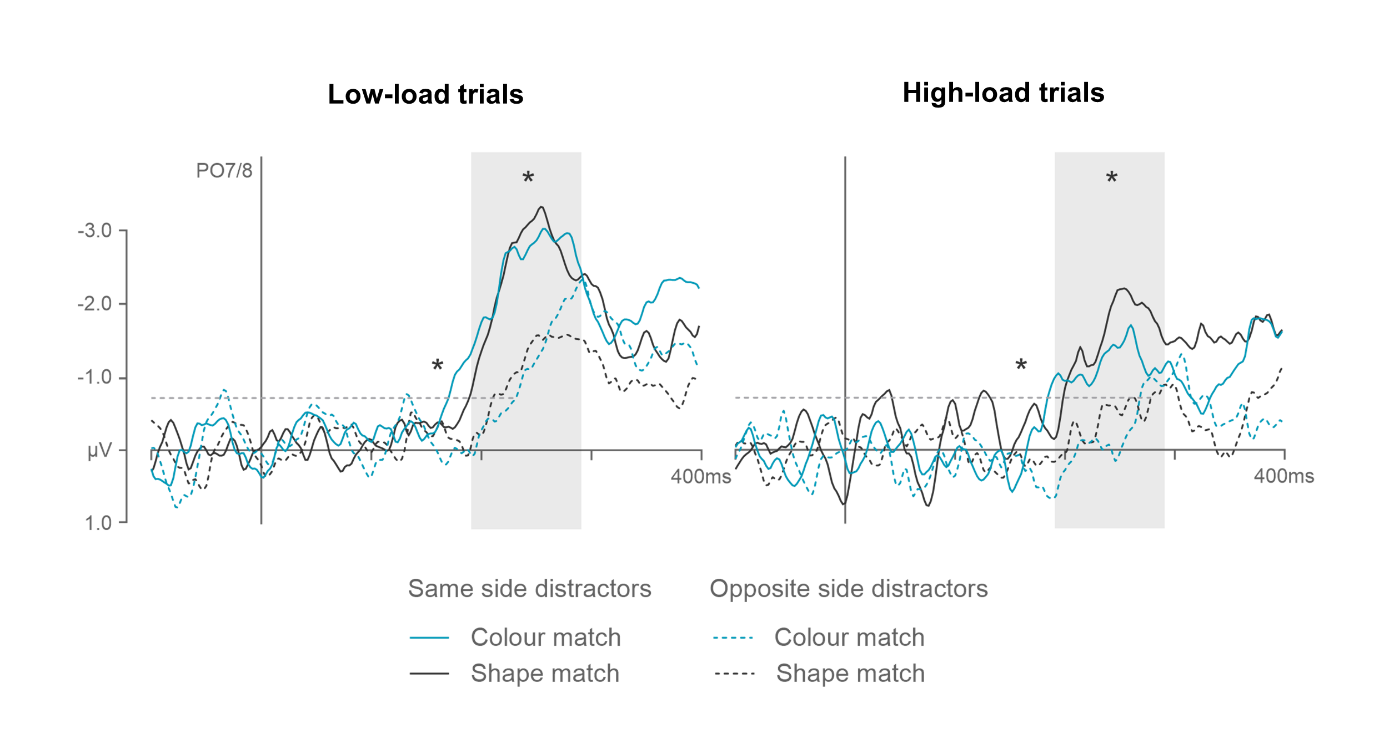


**Figure S2.** N2pc difference waves in low- and high-load trials of the conjunction task in Experiment 2, separately for trials in which partially matching distractors were presented in the same or opposite hemifield of the target. N2pcs are shown separately for distractors that matched the target colour or shape, respectively. The shaded areas mark the N2pc time window (190-290ms after search display onset) and the dashed lines indicate the onset latency criterion (-0.7μV). Asterisks mark statistically reliable amplitude (asterisks above amplitudes) and latency differences (asterisks above onset criterion) between N2pcs triggered in response to targets with same or opposite side distractors.
