# Supplementary material for: The Guidance of Attentional Selectivity in Visual Search Is Always Feature‐Based: Behavioral and Electrophysiological Evidence From Feature and Conjunction Search Tasks
Source: Psychophysiology. 2025 Oct 22;62(10):e70169. doi: 10.1111/psyp.70169 (PMC12541687; doi:10.1111/psyp.70169)
Supplement: Supplementary file 4 — Table S2: Mean reaction times (RTs; in milliseconds) and error rates (ER; % of trials) measured in low‐ and high‐load trials of the color, shape, and conjunction tasks in Experiment 2. Brackets contain standard deviations. [file PSYP-62-e70169-s003.docx]

**Table S2.** Mean reaction times (RTs; in milliseconds) and error rates (ER; % of trials) measured in low- and high-load trials of the colour, shape, and conjunction tasks in Experiment 2. Brackets contain standard deviations.
